# Developing a Health System Literacy Measure for Chinese Immigrants in Canada: Adapting the HLS_19_–NAV Scale

**DOI:** 10.3390/healthcare13192410

**Published:** 2025-09-24

**Authors:** Anh Thu Vo, Ying Cao, Lixia Yang, Robin Urquhart, Yanqing Yi, Peizhong Peter Wang

**Affiliations:** 1 Faculty of Medicine, Memorial University of Newfoundland, St. John’s, NL A1B 3V6, Canada; 2 The Centre for New Immigrant Well-Being (CNIW), Markham, ON L3R 6G2, Canada; 3 Department of Psychology, Toronto Metropolitan University, Toronto, ON M5B 2K3, Canada; 4 Department of Community Health and Epidemiology, Dalhousie University, Halifax, NS B3H 1V7, Canada; 5 Cancer Outcomes Research Program, Nova Scotia Health Authority, Halifax, NS B3S 0H6, Canada; 6 Department of Surgery, Nova Scotia Health Authority, Halifax, NS B3H 2Y9, Canada; 7 Dalla Lana School of Public Health, University of Toronto, Toronto, ON M5T 3M7, Canada

**Keywords:** healthcare navigation, health system literacy, health literacy, psychometric validation, factor analysis, Chinese population, immigrants, Canada

## Abstract

**Background:** Health system literacy is crucial for immigrants to navigate health care systems and access necessary services. Little is known about how well immigrants understand and use the healthcare system in Canada. This study aimed to adapt and validate a health system literacy scale for the Canadian context (HSL-CAN). **Methods:** A cross-sectional online survey was conducted from March 11 to July 19, 2024, among Chinese individuals aged 30 or older who have lived in Canada for at least 6 months. The HSL-CAN was developed through a literature review, patient and provider consultation, and adaptation of the European Health Literacy Population Survey 2019–2021 for navigational health literacy measurement (HLS_19_–NAV) and was then translated into simplified and traditional Chinese. Content validity was evaluated via stakeholders’ feedback, and structural validity was evaluated via exploratory and confirmatory analyses (EFA/CFA). Convergent and discriminant validity, as well as known-group validity, were tested using correlations with the HLS_19_-SF12, ANOVA (or t-test), and effect size. Internal consistency was measured with Cronbach’s alpha coefficient and composite reliability. **Results:** Initially, HSL-CAN contained 25 items developed using a five-point Likert response scale. Some minor revisions were made according to the stakeholders’ feedback (n = 12). Five redundancy items were removed based on the EFA. CFA supported a one-factor model with good fit indices (CFI = 0.960, TLI = 0.955, SRMR = 0.033, RMSEA = 0.025),$\;\chi^{2}$/*df* = 1.41). The scale showed a solid internal reliability (Cronbach’s alpha = 0.81; composite reliability = 0.812). The HSL-CAN is highly correlated with the “health care” construct but lowly with the “health prevention and promotion” construct of HLS_19_–SF12. Known-group validity showed large mean differences by education, income, and non-cancer chronic comorbidities and small to moderate mean differences by gender, age groups, employment status, self-rated health, and assistance needed to see a healthcare provider. **Conclusions:** The HSL-CAN is the first validated instrument to evaluate health system literacy in the Chinese population in Canada. Given strong validity and reliability, the instrument can be useful for research and practice, although further refinement is recommended before using this scale on the general population in Canada.

## 1. Introduction

The “silos phenomenon” in healthcare delivery in the Organization for Economic Co-operation and Development (OECD) countries refers to a system of separate care settings governed at different government levels and operated under different budgetary regimes [1]. Patients need to navigate and interact with different providers across uncoordinated departments such as hospitals, primary care clinics, pharmacies, homecare, and insurance companies to access care they need [2]. Some navigational tasks can include understanding how the system works, knowing the roles of different healthcare professionals, being aware of available services and supports, and applying this knowledge to find services, make treatment decisions, or advocate for oneself when needs have not been met [3,4].

These tasks can be challenging for many of them, particular those who are unfamiliar with the healthcare system, those with multiple disease conditions, or those from low socio-economic backgrounds with limited health literacy [3,4,5]. To navigate such a complex and fragmented health system successfully, patients and their caregivers must take proactive roles to navigate the health system [3] or possess adequate health literacy, defined as “the degree to which individuals have the capacity to obtain, process, and understand basic health information and services needed to make appropriate health decision” [6,7].

Health literacy plays a crucial role in navigating the healthcare system [7]. However, more than half of people in developed countries, including European countries [8], Australia [9], and the US [10], have inadequate health literacy. Patients with low health literacy often struggle to navigate the healthcare system, with difficulties in understanding health insurance processes, choosing appropriate health plans, and communicating with healthcare providers [6,8,9]. Similarly, about 60% of adult Canadians struggle with health literacy to navigate the healthcare system [8], and nearly a quarter reported difficulty finding where to access care [9]. Challenges in navigating the complex health system are particularly pronounced for older adults [11], those with multiple comorbidities [12], and immigrants [12].

However, health literacy assessment relies on the instruments used [13]. Several instruments, such as the Test of Functional Health Literacy in Adults (TOFHLA) [14,15], the Newest Vital Sign (NVS) [16], the Rapid Estimate of Adult Literacy in Medicine (REALM) [17], and the Health Literacy Questionnaire (HLQ) [18,19], are commonly used to measure general health literacy for the population. These instruments primarily assess reading, pronunciation, and numeracy skills [14,15,16,17,18,19], as well as incomprehensively encompass the concept of health system literacy [4,20]. Thus, these instruments may fail to measure health system literacy adequately [14,15,16,17,18,19,20]. To address this gap, the M-POHL Network developed the HLS_19_-NAV instrument that was designed to better measure navigational health literacy [20].

To date, the HLS_19_-NAV has been validated in many European countries [20,21,22] but not yet within the Canadian healthcare context. Our study aims to develop a Health System Literacy scale for Canada (HSL-CAN), adapted from the HLS_19_-NAV, and to assess its psychometric properties using the data collected through an online survey in Chinese populations in Canada. This study population was selected because the Chinese population accounts for 4.7% of the Canadian population and represents a broad range of generational statuses from first-generation to third-generation or beyond [23]. This instrument can enhance our understanding of Chinese patients’ ability to navigate the Canadian healthcare system. While focused on Chinese Canadians, this instrument can also offer a foundation for future instrument validation and applications across diverse populations in Canada.

## 2. Methodology

### 2.1. Data Source

This study followed the COSMIN Reporting Guideline for studies on measurement properties to ensure that all measurement properties of our scale, as well as risks of bias, are reported transparently [24] (see Supplementary S1). The data for analysis came from an online cross-sectional survey conducted between 11 March and 19 July 2024, among adult Chinese individuals aged 30 or older who have lived in Canada for at least 6 months. The survey aimed to explore barriers to medical care utilization among Chinese immigrants in Canada during the COVID-19 pandemic. It included questions on socio-demographics, medical history, medication use, health literacy, and cancer screening. We used the age cut-off of 30 for three main reasons. First, young adults generally have limited interactions with the healthcare system. New immigrants in this age group tend to be healthier than their counterparts due to “healthy immigrant effect” and thus report the lowest healthcare utilization [25,26]. Research also showed that young individuals aged 20 to 29 often avoid seeking care [25] or prefer using emergency care for non-urgent health conditions [25,27]. Second, healthcare-seeking behavior among young adults is often influenced by their parents, who determine whether they should seek care or act as navigators or advocators for their children in the healthcare system. This is true in Chinese young adults due to strong familial ties [27]. Given limited experiences in healthcare systems, inclusion of this population can result in a high chance of response bias when we use online survey modes in data collection. Moreover, because this study was embedded within a larger umbrella survey with multiple objectives, we had to adhere to the inclusion criteria of the host survey that includes participants aged 30 or older.

The online survey was developed and delivered through the Qualtrics platform provided by Memorial University of Newfoundland and was promoted through various social media platforms, including WeChat, websites of collaborative Chinese community organizations, Facebook groups, and other approaches (e.g., emails). Participants were required to give online informed consent before beginning and completing the survey. Although Qualtrics recorded all responses, including incomplete or unsubmitted ones, only those who selected “agree to submit” were included in the analysis. Participation was voluntary and anonymous. Identifying variables such as IP address, WeChat ID, or email were not included in data analysis.

Of the 797 respondents, a total of 681 eligible individuals were included for data analysis. We excluded 111 respondents because these participants either declined to submit or did not indicate whether they agreed to submit the survey, although Qualtrics recorded these latter cases. According to COSMIN criteria, a sample with a size of 7 times the number of items in the investigating model and at least 100 responses is considered very good for structural validity using factor analysis [28]. Therefore, our sample size of 681 is suitable for analysis.

### 2.2. A Description of Study Sample

Among the 681 participants, 97.80% were Canadian citizens or permanent residents, 55.07% were women, and half (50.07%) were aged between 50 and 64. A majority of participants lived in Ontario (58.15%), while the remainder lived in other provinces across Canada, including Quebec (12.19%), British Columbia (13.66%), Alberta (6.75%), Saskatchewan (1.03%), Manitoba (2.79%), and Atlantic provinces (5.43%).

About 78.85% of participants were born in mainland China, while others were born in Hong Kong (8.96%), Taiwan (9.54%), and Canada (2.35%). Most respondents (76.65%) have lived in Canada for at least 10 years. Approximately 97.7% participants obtained a college or university degree (45.37%) or postgraduate education (52.28%), while only 2.35% had completed high-school only or other types of education. Moreover, 86.34% were married, and 62.70% were either employed (45.08%) or self-employed (17.62%). Participants with a total household income under CAD 60,000 accounted for 13.51%, while 78.71% had a household income at least CAD 60,000. Nearly one quarter (24.08%) of participants reported fair or poor health, 56.83% had at least one non-cancer chronic comorbidity and 18.36% had been diagnosed with a cancer, and 45.67% respondents reported regularly using traditional Chinese medication. While most participants (91.92%) reported having a general practitioner when they needed care, 44.35% needed assistance to access healthcare providers (see Table 1).

### 2.3. Measures of Health Literacy

Ten items of the HLS_19_–NAV scale (D1, D5, D7, D9–D11, and D13–D16) were used in our new scale. The original 12-item scale was developed as part of the European Health Literacy Population Survey 2019–2021 (HLS_19_) [4,14]. Item development was conducted through definitions, conceptual frameworks, and existing tools associated with navigating the healthcare system [4]. The first version was developed in German and translated into English [4]. Twelve items assess patients’ abilities in accessing information (3 items), understanding information (3 items), judging information (3 items), and using the information to communicate with healthcare providers and navigate the healthcare system to access care they need [4] (see Supplementary S2). Each item of the HLS_19_–NAV has 4 response categories: “4—very easy”, “3—easy”, “2—difficult”, and “1—very difficult”.

The adapted scale—the HSL-CAN—has five response categories: “1—extremely difficult”, “2—somewhat difficult”, “3—neither easy nor difficult”, “4—somewhat easy”, and “5—extremely easy”, which can potentially improve its psychometric characteristics [29] (see Supplementary S3).

#### 2.3.1. Item Development

Along with items from the HLS_19_–NAV, the HSL-CAN scale was developed through literature review on patients’ and caregivers’ experiences with navigating the health system and constructs from existing tools regarding healthcare system navigation based on Griese et al. (2020)’s approach [4]. We also consulted a patient advisory committee with 07 members, including three cancer patients, a physician, two community service providers, and a researcher. The scale was translated from English into simplified Chinese and traditional Chinese versions and translated back into English. Any discrepancies were resolved through discussion between Y.C. and A.T.V.

#### 2.3.2. Content Validity

The pre-survey was sent to 12 colleagues who had worked in academia and the Canadian healthcare system. They were asked to provide their feedback on the understandability and content of the survey. Y.C. and A.T.V. revised the items to ensure all feedback was appropriately addressed. In addition, we also conducted a post-survey validation with our patient advisory committee with five members participating (one physician, one researcher, one community service provider, and two patient partners). This post-survey validity aims to confirm the suitability of the final instrument after removing some deemed inappropriate items based on statistical analyses. If there were any discrepancies among stakeholder input and results from the statistical analyses, we reviewed all items retained to ensure that their concerns were addressed.

### 2.4. Statistical Analyses

#### 2.4.1. Structural Validity Test

- Exploratory factor analysis (EFA)

The EFA was utilized to examine the internal consistency of items and to identify the numbers of factors and their associated items [30]. The EFA explores the underlying structure of the scale when being adapted to the Chinese population in the Canadian health system [31]. To start with, the EFA can assist our understanding of possible cultural difference in the adaption of the scale [32]. This technique determines how strongly an item loads onto an underlying factor indicated by factor loading values, where a loading value of >0.3 or >0.4 indicates moderate [33] or good correlation [34], respectively. This study used the cut-off factor loading value of ≥0.4.

Before extracting factors, the suitability of data for factor analysis was examined using Kaiser–Meyer–Olkin (KMO) to assess sampling adequacy and Bartlett’s test of sphericity to examine whether correlations among any different items are statistically significant [30]. The KMO test values are between 0 and 1, with values over 0.7 indicating adequate sampling for factor analysis [24]. A significant *p*-value < 0.05 from Bartlett’s test of sphericity indicates the data are suitable for factor analysis [30].

Weighted least square based on a poly-choric correlation matrix for factor extraction was applied given an ordinal, rather than normal, distribution of the data [35,36]. We also used parallel analysis to determine the number of factors to retain by comparing eigenvalues with random order eigenvalues [35,37]. Factors with actual eigenvalues > 1 and surpassing the random order eigenvalues were retained [35,37]. An orthogonal varimax rotation was used to maximize high item loadings and minimize low item loadings, producing a more interpretable and simplified solution if more than one factor was retained from parallel analysis [37]. Moreover, communality values, referring to the percentage of an item’s variance explained by the factor, were reported [35]. Multicollinearity was examined using the variance inflation factor (VIF), where a value above 5 or 10 [38] or correlations between items > 0.9 [35] indicates multicollinearity.

b. Confirmatory factor analysis (CFA)

The CFA was used to test the model in EFA with items retained and to evaluate a goodness of fit of the model. The restricted CFA was fitted with no cross-loadings or correlated residuals, and parameters were estimated using diagonally weighted least square (WLSMV) [39,40]. This study reported goodness-of-fit statistics, including the Tucker–Lewis index (TLI), the comparative fit index (CFI), the root mean square error of approximation (RMSEA), and the standardized root mean square residual (SRMR). Values of ≥0.95 for TLI and CFI and ≤0.08 for SRMR and RMSEA were considered for good model fit [41]. The Chi-square statistic ($\chi^{2}$) was not reported, because of the sensitivity to sample size [41]. Instead, $\chi^{2}$/*df* were reported since $\chi^{2}$ and *df* increase as a function of the number of variables [41]. A $\chi^{2}$/*df* ratio < 3.00 indicated an acceptable fit [41].

In addition to validating the scale’s structure using EFA, we assessed other structures of the scale based on the judgment of item contents: (1) the underlying dimension of health literacy (understand, find, assess, and use) and (2) the underlying dimension of health system navigations (system level vs. organizational level). We developed several different models: a unidimensional model obtained from EFA; a correlated-factor model; a bifactor model; and a hierarchical model based on assumptions about the correlation between items, dimensions (sub-factors), and general factors. The bifactor model assumes that these dimensions are not correlated with the general factor (health system literacy), and the high-order model assumes that these four factors are correlated the general factor [42]. CFA is a good technique to evaluate the goodness of fit among models [42,43]. We used the CFA model to compare improved good fit indices among models. CFA analysis enables us to explore potential structure of the scale along with the structure obtained from EFA. Additionally, if a multidimensional structure is applicable, the bifactor and high-order models allow us to examine whether an overall score and sub-scores are required to report [42]. We used the entire sample for CFA analysis because this approach helps identify discrepancies in results from EFA and CFA in the same population [44].

#### 2.4.2. Reliability

Cronbach’s alpha coefficient and composite reliability were used to calculate the internal consistency and reliability of the scale and subscales if applicable. Cronbach’s alpha is the most commonly used measure of internal consistency based on correlation of items, with a value ranging between 0 and 1 [45]. Composite reliability produces a more accurate estimate of the reliability than Cronbach’s alpha because it allows factor loadings to vary while these loadings are constrained to be equal in Cronbach’s alpha [46]. A value of Cronbach’s alpha or composite reliability between 0.60 and 0.70 is considered acceptable, and values above 0.7 indicate good reliability [45]. However, values > 0.9 may suggest item redundancy and are typically not desirable [45].

#### 2.4.3. Cross-Validation CFA Models

Once the optimal model that obtained good fit indices and acceptable reliability was selected, we validated the goodness-of-fit model across subsamples of the dataset using k-fold cross-validation. In k-fold cross-validation, the entire dataset was divided into k number of folds [47]. The process was repeated k times with one-fold used as the test set and the remaining (k-1) folds used as the training test in each iteration [47]. The value of k is generally recommended from 5 to 10, but the choice depends on the sample size of dataset [47]. In this study, we set k = 3, training the model on two-fold and evaluating it on the one-fold in each iteration.

#### 2.4.4. Construct Validity

This study used a confirmatory factor analysis to examine factor correlation between measures of similar (convergent validity) and different constructs (discriminant validity also known as divergent validity) [48]. The 12-item short form Health Literacy Scale (HLS-SF12) developed by Tuyen V. Duong for Asian individuals aged 15 or older [49] was used for this purpose. The original scale has three dimensions: “health care” (4 items), “disease prevention” (4 items), and “health promotion” (4 items), with each item rated on a 4-point Likert scale (1, very difficult; 2, difficult; 3, easy; 4, very easy) [49] (see Supplementary S4). Duong et al. (2019) reported a Cronbach’s Alpha coefficient of 0.49 to 0.72 for the health care subscale, 0.64 to 0.77 for the disease prevention subscale, and 0.63 to 0.81 for the health promotion subscale [49]. Regarding the HLS-SF12 scale, our study found that the HLS-SF12 obtained a goodness-of-fit model with two factors: “health care” and “prevention and health promotion” (CFI = 0.95, TLI = 0.93, RMSEA_90%CI_ = 0.04 (0.02–0.05), SRMR = 0.05, $\chi^{2}$/*df* = 96.146/53 = 1.81). We found a Cronbach’s alpha coefficient of 0.53 for the “health care” and 0.63 for the “prevention and health promotion” subscale (see Supplementary S4).

Known-group validity was assessed by comparing mean differences by sociodemographic characteristics using *t*-test, one-way ANOVA, post hoc tests (Tukey’s HSD for homogeneous variances and Games–Howell for heterogenous variances), and Hedge’s effect size. The instrument score was calculated by the proportion of items with valid responses choosing “easy” or “very easy” with at least 80% of items containing valid responses [50]. While this dichotomous score may prevent assumption of equal intervals in ordinal scales, it can result in lost information due to dichotomization [51]. The polychotomous score can be used, as it aligns with previous validation studies of HLS-EU and makes statistical analysis more convenient, especially when data is assumed to follow a nearly normal distribution [20,51]. However, this type of score can be inflated by extreme response styles [51]. In this study, the polychotomous score was calculated using the formular in Tuong V. Duong et al. (2019): score = (raw mean − 1) × (100/4), where raw mean is the mean of all valid responses, 4 is the range of the mean, and 100 is the maximum standardized score [52]. In both approaches, the range of the score is between 0 and 100. This study used dichotomous and polytomous scores for known-group validity to examine whether these two score types produce comparable results.

The health system literacy score was considered as the dependent variable, while other variables were treated as independent variables: gender (men vs. women), age group (under 50, 50–64, vs. 65 or older), education (college/university degree, postgraduate—master/Ph.D., vs. other), income (under CAD 60,000, CAD 60,000–89,999, vs. ≥CAD 90,000), length of stay in Canada (<5 years, 5 to less than 10 years, vs. ≥10 years), marital status (married vs. others), current employment status (employed, self-employed, vs. others), self-rated health (excellent to very good, good, vs. fair to poor), number of non-cancer chronic comorbidities (none, 1, 2, >2, vs. do not know), diagnosed with cancer (yes vs. no), having a general practitioner/family physician (yes vs. no), and assistance to see an HCP (yes vs. no). Effect size was measured using Hedges’ g for standardized difference in means, with Hedges’ g values of 0.2 to <0.5 as a small effect, 0.5 to <0.8 as a medium effect, and ≥0.8 as a large effect [53].

#### 2.4.5. Missing Data

The amount of missing data within HLS-CAN items was very small, ranging from 0.15% to 1.47% (see Supplementary S3). Hence, a listwise deletion was used to address missing data.

SAS 9.4 was used for data cleaning, and R programming was used for data analysis. The following R-packages were used in this study, including “psych” for exploratory factor analysis and the reliability test; “lavaan”, “semTools”, and “semPlot” for confirmatory factor analysis and path diagram [54]; “effsize” for calculating the effect size of mean differences [55]; and “caret” for cross-validation evaluation [56].

## 3. Results

### 3.1. Item Development

Along with 10 items from the HLS_19_–NAV, we developed 14 new items based on literature review and consulting with the patient advisory committee. Following the Griese et al. (2020) approach, all items were designed to cover four dimensions of health literacy: understand (five items), find (eight items), assess (four items), and use (eight items) [4,20]. Additionally, the potential subscales based on the navigational health system conceptual frameworks include the system-level subscale (five items) and the organizational-level subscale (seven items) [20].

Table 2 presents items included in the HSL-CAN scale. We modified item D10 emphasizing the understanding of rather than finding out about their rights, as a patient in the healthcare system (item 5 in HLS-NAV). Item D11 was modified to highlight the ability to assess the quality of a particular health service rather than to find information about the quality of a particular health service. In our adapted scale, 25 items captured four dimensions of the health literacy concept, understand (five items), find (six items), assess (six items), and use (eight items), as well as covered three levels of navigational health system with organizational and interactional levels combined as the organizational level, including the system level (7 items) and the organizational level (18 items).

### 3.2. Content Validity

The pre-test survey was conducted by emailing it to 12 colleagues. They were asked to review and provide their feedback on the understandability and content of the survey. We received some feedback regarding wordy questions, unclear terminology (e.g., insurance benefits), and concerns that item D22 was not suitable in the Canadian context. There were some minor revisions based on the stakeholders’ feedback (see Table 2).

### 3.3. Translation

The English version of the survey was translated into Chinese simplified and traditional versions by Y.C. and verified by N.L., then translated back into English using ChatGPT 4.0 and verified by A.T.V. A few variations between the back-translated version and the original version were resolved through discussion.

### 3.4. Exploratory Factor Analysis

**Figure 1:**
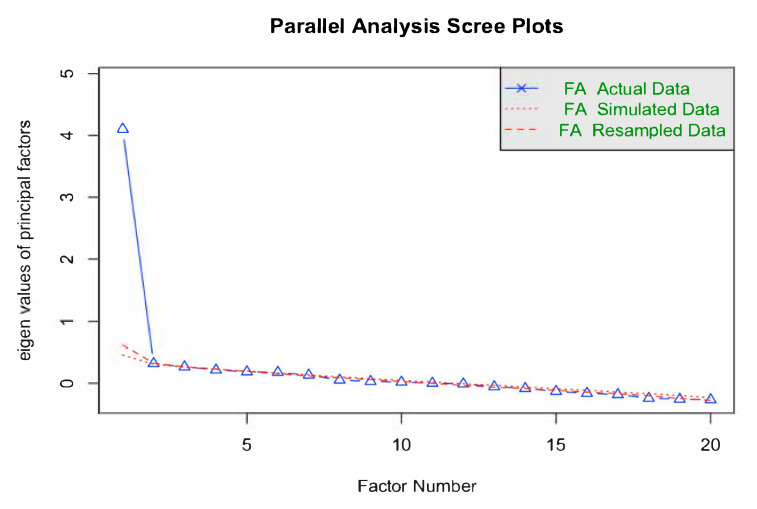
Parallel analysis and scree plot for exploratory factor analysis of HSL-CAN scale.

The overall KMO values were 0.9, and Bartlett’s test of sphericity was significant (*p* < 0.05), indicating an adequate sampling and significant correlation among different items. This suggests that data are suitable for factor analysis. The results from parallel analysis and weighted least squares suggest that the number of factors is one. Additionally, the Scree plot illustrated a steep drop from the first factor to the second factor, indicating a one-factor structure of the scale (see Figure 1). Five items (D6, D8, D18, D20, and D23) with loading under 0.40 were removed (see Table 3). A repeated KMO test and Bartlett’s test of sphericity for the dataset of 20 remaining items indicated the five-items-excluded dataset is suitable for factor analysis, with overall KMO values of 0.9 and a significant Bartlett’s test of sphericity (*p* < 0.05). Moreover, the VIF values ranged from 1.18 to 1.26, and no inter-item correlations exceeded 0.8, indicating that multicollinearity among items was not present (see Supplementary S5).

### 3.5. Confirmatory Factor Analysis and Internal Reliability

Based on the CFA output, the bifactor two-factor model and bifactor four-factor model did not support the theoretical models that grouping factors are uncorrelated with each other and with a general factor. Additionally, standard errors could not be estimated, and some estimated variances were negative, indicating the hierarchical two-factor model is ill-conditioned. Therefore, four models, the one-factor model derived from EFA, the correlated two-factor model, the correlated four-factor model, and the hierarchical four-factor model, were compared to determine which model best fit the data.

All goodness-of-fit indices yielded from four models are within acceptable cutoff values: CFI ≥ 0.95 (CFI_one-factor_ = 0.99, CFI_correlated two-factor_ = 0.99, CFI_correlated four-factor_ = 0.99, and CFI_hierarchical four-factor_ = 0.99), TLI ≥ 0.95 (TLI_one-factor_ = 0.99, TLI_correlated two-factor_ = 0.99, CFI_correlated four-factor_ = 0.99, and CFI_hierarchical four-factor_ = 0.99), RMSEA_(90%CI)_ ≤ 0.08 (RMSEA_one-factor_ = 0.03_(0.02–0.03)_, RMSEA_correlated two-factor_ = 0.03_(0.02–0.03)_, RMSEA_correlated four-factor_ = 0.02_(0.02–0.03)_, and RMSEA_hierarchical four-factor_ = 0.02_(0.02–0.03)_), SRMR ≤ 0.08 (SRMR_one-factor_ = 0.04, SRMR_correlated two-factor_ = 0.04, SRMR_correlated four-factor_ = 0.04, and SRMR_hierarchical four-factor_ = 0.04), and $\chi^{2}$/*df* < 3.00 ($\chi^{2}$/*df*_one-factor_ = 1.75, $\chi^{2}$/*df*_correlated two-factor_ = 1.76, $\chi^{2}$/*df*_correlated four-factor_ = 1.63, and $\chi^{2}$/*df*_hierarchical four-factor_ = 1.61). However, improved fit indices were found in models with four factors compared with those with one or two factors (see Supplementary S6).

Despite the improved goodness of fit in models with four factors, the factors were highly correlated with each other. In the correlated two-factor model, the inter-factor correlation was 0.97, indicating that two factors shared a large proportion of variances or probably measured the same construct. Similarly, the inter-factor correlations in the correlated four-factor model ranged from 0.84 to 0.94, and the correlation between a general factor and four factors in the hierarchical four-factor models ranged from 0.89 to 0.99. These findings strongly suggested that all four factors are measuring the same underlying construct. In other words, a unidimensional model may be more appropriate.

Moreover, the adapted scale demonstrated a good internal consistency with an overall Cronbach’s alpha coefficient of 0.82 and an overall composite reliability value of 0.82 across models. However, the internal reliability subscales are only acceptable, with Cronbach’s alpha coefficients of 0.62 for factor 1 and 0.76 for factor 2 in the two-factor model. In contrast, the subscales of the four-factor models had an unacceptable internal consistency with Cronbach’s values and composite reliabilities falling below 0.6.

### 3.6. Cross-Validation

CFA was conducted on the one-factor model using a sub-sample (n = 227) from the entire dataset (n = 681) using the three-fold cross-validation procedure. The CFA model obtained a good fit across the cross-validation samples where all fit indices fell within the acceptable ranges, although these indices were not as strong as those in the model with the entire model (see Table 4). This suggested that CFA goodness-of-fit indices may be influenced by the sample size.

### 3.7. Construct Validity

#### 3.7.1. Convergent and Discriminant Validity

The two subscales of the HLS-SF12 were used to evaluate convergent and discriminant validity in this study. We hypothesized that our construct was more likely related to the “health care” construct and was not closely related to the “prevention and health promotion” construct. The CFA outputs showed a strong correlation with the “health care” subscale (r = 0.79, *p* < 0.05), supporting a good convergent validity. In other words, both measures may measure a similar construct. Moreover, our scale demonstrated a good discriminant validity, as evidenced by a lower correlation coefficient between our scale and the “prevention and health promotion” subscale (r = 0.21, *p* = 0.002), suggesting that two measures may be measuring distinct constructs (see Supplementary S7).

#### 3.7.2. Known-Group Validity

Table 5 and Supplementary S8 present differences in mean scores of health system literacy across socio-demographic characteristics and their effect sizes. No significant difference in mean health system literacy scores and uncertain or very small effect sizes were found for length of stay in Canada and marital status (*p*-value > 0.05), with dichotomous scores and polytomous scores.

Significant differences in mean health system literacy scores with both dichotomous and polytomous were observed in gender, age group, highest level of education, income level, number of non-cancer chronic comorbidities, and self-rated health. Among these groups, the education group, income level, and number of non-cancer chronic comorbidities have the moderate to high effect sizes (Hedges’s g ≥ 0.5). Participants with a postgraduate degree (master/Ph.D.) reported the highest health system literacy score, particularly when compared with those with the lowest education level (ES = 0.73 in dichotomous scores; ES = 1.05 in polytomous scores).

The highest health system literacy scores were observed among individuals with a total annual household income of CAD 60,000 to CAD 89,999, followed by those with total income ≥ CAD 90,000 (ES = −0.72 in dichotomous scores; ES = −0.71 in polytomous scores). Conversely, those with a total annual household income of <CAD 60,000 reported the lowest score (ES = −0.84 in dichotomous scores; ES = −0.96 in polytomous scores). Non-cancer chronic comorbidity status was significantly associated with the health system literacy score. The highest scores were found in participants with at least two comorbidities, while the lowest score was observed in those uncertain about their health conditions, with effect sizes ranging from 0.96 to 1.12 (dichotomous scores) and 0.88 to 1.09 (polytomous scores).

In contrast, differences in mean literacy scores among age groups, genders, and self-rated health had small to moderate effect sizes. Individuals aged under 50 reported the highest score, followed by those aged 50 to 64 and 65 or older, with small to moderate differences ranging from 0.29 to 0.76 (dichotomous scores) and 0.28 to 0.72 (polytomous scores). Men had a higher mean score compared with women, with a small effect size (ES = 0.23). Individuals who rated their health as excellent to very good had the highest health system literacy score, whereas those with good self-rated health had the lowest score (ES = 0.27).

However, inconsistent findings between dichotomous scores and polytomous scores were found for current employment status, cancer diagnosis, having a general practitioner/family physician, and the need for assistance to see an HCP. Employed or self-employed individuals, as well as those who needed assistance to access an HCP, reported a higher mean health system literacy score (both dichotomous score and polytomous scores) than their counterparts, but these differences were small and only significant for dichotomous scores. Similarly, cancer patients reported lower mean scores than those without cancer; however, only the difference in dichotomous scores was significant, and its true effect size was uncertainty (−0.17, 95% CI: −0.37–0.02). In contrast, participants who have a general practitioner/family physician had higher scores than those without having HCPs, but a significant difference was found only in the polytomous score, with a small effect size (ES = 0.49).

### 3.8. Patient and Public Engagement

Based on insightful feedback from our patient advisory committee, most members agreed that the final scale is relevant and effectively captures the construct of health system literacy. The scale reflects patient experiences related to navigating the health system. One patient partner recommended retaining items D6 (How easy is it for you to find a family doctor or primary healthcare provider near you?) and D8 (How easy is it for you to book an appointment with a healthcare provider?). However, we considered that D7 (How easy is it to understand how to get an appointment with a particular health service?) may cover the content of D6 and D8.

The committee found the scale generally clear and easy to understand. However, some medical jargon terms (e.g., healthcare system, services, providers, insurance, and “right person”) should be clarified or illustrated with simple examples. To minimize a high non-response rate due to questions with more words, we kept our online survey more concise, although this limited our ability to define the technical terms in detail. A few suggestions for improving the scale can include rewording D5, using simpler response categories (e.g., very difficult, difficult rather than extremely difficult, and somewhat difficult), and providing examples to clarify the technical terms.

## 4. Discussion

The study presents the validation of psychometric properties of the HSL-CAN scale among a Chinese immigrant population aged 30 or older in Canada. Our findings addressed literature gaps about a need for understanding how well Chinese populations understand and use the Canadian healthcare system. The 20-item scale included 10 items adapted from the HLS_19_-NAV [4] and 10 newly developed items. These items cover four dimensions of health literacy, understanding (five items), finding (six items), accessing (five items), and using (four items), as well as encompass various aspects of health literacy across multiple levels of health system navigation to identify areas where healthcare providers and decision-makers should focus their attention.

Exploratory factor analysis and confirmatory factor analysis indicated that the 20-item scale obtained goodness of fit within a one-factor construct. This finding aligns with the results of the Griese et al. (2022) study, which reported acceptable fit indices for a single-factor model [4,20]. In contrast, one study conducted in Russia by Drapkina et al. (2025) indicated that HLS_19_-NAV-RUS demonstrated improved fit values in a two-factor CFA model compared with the one-factor CFA model [22]. This difference suggests cultural differences that might influence cross-cultural adaptation of the HLS_19_–NAV scale across countries with different healthcare systems [22,57].

The HLS_19_-NAV scale evaluated how well patients understand and navigate the healthcare system. The structure of a healthcare system (e.g., decentralized vs. centralized healthcare systems) might contribute to variation in the factor structure of the scale. In decentralized health systems, power, responsibilities, resources, and decisions are typically redistributed to local governments. In contrast, centralized systems typically place these responsibilities in the national/sub-national government [58]. Because the delivery of health services and resources in smaller jurisdictional areas are influenced by organizational operations and policies [59], patients from countries with decentralized health systems (e.g., European countries [60], Canada [61], and USA [62]) must navigate various types of services, healthcare providers, and regulations at multiple levels. As a result, a one-factor scale can reflect a general ability to navigate the healthcare system among populations from decentralized health systems. Conversely, in centralized health system countries (e.g., Russia), regulations and healthcare services that are established by national governments are more uniform across regions [59,63], allowing their patients to distinguish between system-level experiences (understanding how the system works) and organizational-level experiences (navigating healthcare services and providers).

Items in our scale with factor loadings ≥ 0.4 were retained in the model, but the communality values were relatively low, ranging from 0.154 to 0.225. Communality refers to the proportion of each item’s variance explained by the factors [64]. Higher communality values indicate that the variance of the item is more likely to be explained by extracted factors [33]. Acceptable cutoff values for communality should be between 0.25 and 0.4, with an ideal value above 0.6 [65,66]. The low communality values in this study may be explained by several reasons. First, measurement errors can lead to the lower value. Indeed, total variance in exploratory factor analysis is a sum of the variance explained and unique variances [64]. In our study, the risk of measurement error is substantial. Factors contributing to potentially great measurement errors in the survey include cognitive efforts to correctly retrieve the information asked (e.g., recall bias and salience), respondent cooperation (e.g., willing to make effort to provide accurate responses), survey mode [67], ambiguous or complex questions, limited prior experience with the healthcare system, and a lengthy questionnaire [68]. Second, participants may have responded to the question based on their beliefs rather than their actual skills or experiences, which could introduce measurement error. Many Chinese patients often rely on their family members to schedule health appointments, so their responses might not accurately reflect their navigational skills, thereby reducing their correlation with the factor. However, Eaton et al. (2019) argued that stricter values might suggest better goodness of fit of the model, but this must be balanced with retaining an adequate number of appropriate items [69].

The instrument obtained a good reliability and construct validity. Cronbach’s alpha value was 0.81, and composite reliability was 0.812 on Chinese immigrants aged 30 or older in Canada. These results are consistent with the Griese et al. (2022) study, where Cronbach’s alpha and composite reliability values ranged from 0.88 to 0.94 across European countries [20], and with Drapkina et al. (2025), where Cronbach’s alpha value was 0.85 [22]. Moreover, this study also examined convergent and discriminant validity through evaluating the relationship between the HSL-CAN and two constructs of the HLS_19_-SF12 (health care and prevention and health promotion). The construct of HSL-CAN was significantly associated with the HLS_19_-SF12 health care construct but weakly related to the construct of prevention and health promotion, suggesting that all items in the scale measure a specific type of health literacy rather than general health literacy.

Regarding known-group validity, health system literacy was associated with some sociodemographic factors, including gender, age group, highest level of education, income level, self-rated health, number of non-cancer chronic comorbidities, diagnosed with cancer, having a general practitioner for health need, and a need for assistance to access an HCP for either dichotomous scoring or polytomous scoring. Particularly, education group, income level, and number of non-cancer chronic comorbidities showed the largest effect size. Griese et al. (2022) similarly revealed that lower NAV-HL scores were associated with high financial deprivation and older age [20]. Therefore, this scale demonstrates a practical applicability in distinguishing subgroups and evaluating population health system literacy for research purposes.

However, the choice of scoring method may cause inconsistences in results. Significant differences in mean health system literacy scores were found among gender, age group, highest level of education, income level, self-rated health, and number of non-cancer chronic comorbidities for both dichotomous and polytomous scoring. In contrast, significant differences were observed with dichotomous scoring for current employment status, diagnosed with cancer, and need assistance to access an HCP, while differences in scores were significantly found with polytomous scores for having a general practitioner group.

These inconsistencies may result from differences in scoring methods and the natures of the variables. First, dichotomous scoring is more likely to cause information loss since it is sensitive only to responses of “easy” or “very easy” and disregarding other response options. In contrast, polytomous scoring covered all responses of the scale, which might provide more nuanced variances. As a result, significant differences in dichotomous scores were found for variable groups where respondents were more likely to choose “easy” or “very easy”. Second, the nature of the variable groups may impact the results. For example, cancer patients may be less likely to rate navigational health system tasks as “easy” or “very easy” than non-cancer patients because of the known complexity and fragmentations of cancer care systems [70].

Therefore, the choice of scoring method should align with the study’s objectives, whether that is to measure generally an individual’s health literacy score or to assess the perceived ease of navigating a specific health service or process. For example, when the objective is to compare or screen health system literacy in the general populations, dichotomous scoring may be adequate and provides a simpler interpretation. In contrast, for studies aiming to evaluate health system literacy in specific populations (e.g., cancer patients and newcomers), polytomous scoring is highly recommended because it can provide more nuanced information about the difficulty of navigating health systems.

### Strengths and Limitations

The strength of this study lies in the comprehensive testing and reporting of psychometric properties for a health system literacy scale adapted from HLS_19_-NAV among Chinese populations in Canada. This study provides strong evidence supporting the successful cross-cultural adaptation of HLS_19_-NAV into Chinese, within the context of the Canadian health system. Moreover, adherence to the COSMIN reporting guidelines allowed for a rigorous evaluation of the psychometric properties and enhanced the transparency of the findings including identification of potential risks of bias. This can open more opportunities for future studies to improve their qualities of methodological quality, as well as the instrument’s measurement properties.

However, our study has some limitations. First, psychometric properties of the HSL-CAN scale were evaluated only in individuals aged 30 or older, so future studies are required to assess reliability and validity on younger populations under the age of 30. Indeed, studies on this population should be investigated separately from the population aged 30 or older because young individuals are healthier and rarely seek healthcare [25,26]. Given limited experiences in healthcare system, they often rely on their parents for healthcare navigation tasks or avoid seeking care when they have health issues. As a result, their skills and experiences with navigating healthcare systems may be different compared with those of their counterparts.

Second, potential selection bias must be acknowledged in our data collection. A large percentage of individuals in our study held master/Ph.D. degrees, which cannot accurately represent the whole of Chinese Canadians. However, according to the 2021 Census, a higher percentage of recent and established immigrants do have a bachelor’s degree or higher compared with Canadian-born individuals [71]. Additionally, established immigrants from Asian countries (e.g., China, Southern Asia, and Western Asia) and Africa often obtained high education attainment compared with non-immigrant individuals [72]. Moreover, most of our participants came from large provinces such as Ontario and British Columbia, which have more health facilities and resources, which may better support them to navigate the health system compared with those from smaller provinces.

Furthermore, because data were collected through an online survey, individuals who did not have Internet access or mobile devices could not participate in the study. We used an online survey due to its affordability and common use, although this modality posed limitations in interacting with respondents, which prevented us from explaining some difficult terms if participants had queries. To provide a more comprehensive and accurate assessment of health system literacy, future studies should considered other alternative modes of survey administration such as face-to-face interviews or computer-assisted interviewer administration, which allow researchers to interact and clarify vague questions.

Although this study provides at least part of the picture regarding health system literacy in one of largest vulnerable populations in Canada—Chinese Canadians—a comprehensive understanding of the literacy of other populations remains limited. For example, research revealed that South Asian Canadians have low health literacy and face significant challenges accessing the healthcare system and health services [73]. This scale can serve as a useful instrument for future research on these populations.

## 5. Conclusions

Adequate knowledge of the Canadian health system and navigational skills are necessary for patients to navigate the health system and access the care they need. Understanding health system literacy enables healthcare providers and decision-makers to identify what and how healthcare structures and policies require revision to facilitate health navigation processes, as well as to develop support services that enhance health system literacy among patients and caregivers. This study demonstrates that the health system literacy scale adapted from HLS_19_-NAV has obtained suitable structure validity, good construct validity, and high reliability within the Chinese population in Canada. It can serve as a valuable tool for healthcare providers and researchers to assess how well Chinese Canadians understand and use the healthcare system. However, given some limitations existing from the study, the scale should be revised and validated for use with the general Canadian population to ensure reliable and comprehensive assessment of health system literacy.

## Figures and Tables

**Table 1:** Summary of socio-demographic characteristics of the study sample.

Variables	Frequency	Percent
Immigrant status		
Canadian citizenship/PR	666	97.80
Others	13	1.91
Missing	2	0.29
Current province		
Ontario	396	58.15
Quebec	83	12.19
British Columbia	93	13.66
Alberta	46	6.75
Saskatchewan	7	1.03
Manitoba	19	2.79
Atlantic provinces	37	5.43
Gender		
Man	305	44.79
Woman	375	55.07
Missing	1	0.15
Age group		
<50	248	36.42
50–64	341	50.07
≥65	92	13.51
Place of birth		
Mainland China	537	78.85
Hongkong	61	8.96
Taiwan	65	9.54
Canada	16	2.35
Missing	2	0.29
Length of stay (LOS) in Canada		
LOS < 5 years	56	8.22
5 years ≤ LOS < 10 years	102	14.98
LOS ≥ 10 years	522	76.65
Missing	1	0.15
Highest level of education		
College or university	309	45.37
Postgraduate (master/Ph.D.)	356	52.28
Others	16	2.35
Marital status		
Married	588	86.34
Others	93	13.66
Current employment status		
Employed	307	45.08
Self-employed	120	17.62
Others	254	37.30
Religion		
None	268	39.35
Christian	197	28.93
Catholics	96	14.10
Buddhism	64	9.40
Others	53	7.78
Missing	3	0.44
Family total household income		
<CAD 60,000	92	13.51
CAD 60,000 to CAD 89,999	231	33.92
≥CAD 90,000	305	44.79
Missing	53	7.80
Self-rated health		
Excellent/very good	271	39.79
Good	243	35.68
Fair/poor	164	24.08
Missing	3	0.44
Number of non-cancer comorbidities		
No	268	39.35
1	150	22.03
2	177	25.99
>2	60	8.81
Don’t know	19	2.79
Missing	7	1.03
Cancer		
None	551	80.9
Breast cancer	39	5.73
Colorectal cancer	45	6.61
Other cancers	41	6.02
Missing	5	0.73
Use Chinese traditional medication on a regular basis		
Yes	311	45.67
No	361	53.01
Missing	9	1.32
Having a general practitioner/family physician		
Yes	626	91.92
No	54	7.93
Missing	1	0.15
Need assistance to see HCPs		
Yes	302	44.35
No	375	55.07
Missing	4	0.59

**Table 2:** Original items of HSL-CAN scale.

Original Items of HSL-CAN		Revised Items of HSL-CAN	Health Literacy Dimension	Navigational Health System Level
How Easy Is It for You…		How Easy Is It for You…		
D1	to understand information on how the healthcare system works?	to understand information on how the healthcare system works?	Understand	System
D2	to understand roles of different healthcare providers?	to understand what different healthcare providers do?	Understand	System
D3	to understand how long you might have to wait for your health appointment?	to know how long you’ll wait for a health appointment?	Understand	Organization
D4	to find out what healthcare services you’re eligible for, whether through your province’s health plan or private insurance?	to find out which healthcare services you can get, either through your provincial health plan or private insurance?	Find	Organization
D5	to what extent your health insurance covers a particular health service	to figure out how much your health insurance covers for a specific service?	Assess	System
D6	to find a family doctor or primary healthcare provider in your area?	to find a family doctor or primary healthcare provider near you?	Assess	Organization
D7	to understand how to get an appointment with a particular health service?	to understand how to get an appointment with a particular health service?	Understand	Organization
D8	to book an appointment with a healthcare provider?	to book an appointment with a healthcare provider?	Use	Organization
D9	to find the right person to talk about your health concern within a healthcare institution?	to find the right person to talk about your health concern within a healthcare institution?	Find	Organization
D10	to find out your rights as a patient or user of the healthcare system?	to understand your rights as a patient or user of the healthcare system?	Understand	System
D11	to assess the quality of a particular health service?	to assess the quality of a particular health service?	Assess	Organization
D12	to judge whether a health service will meet your expectation and needs in case of a health problem?	to assess whether a health service will meet your expectations and needs in case of a health problem?	Assess	System
D13	to find support options that help you navigate the healthcare system?	to find support options that help you navigate the healthcare system?	Find	Organization
D14	to decide a particular health service (e.g., choose from different hospitals)?	to decide a particular health service (such as choose from different hospitals)?	Use	Organization
D15	to be confident standing up for yourself if your healthcare does not meet your needs?	to stand up for yourself if your healthcare does not meet your needs?	Use	Interaction
D16	to be confident talking with your healthcare providers and making decision together?	to talk with your healthcare providers and make decision together?	Use	Interaction
D17	to find information on preventive health services (like screenings or vaccinations)?	to find information on preventive health services (such as screenings or vaccinations)?	Find	Organization
D18	to access preventive health services (like screenings or vaccination)?	to access preventive health services (such as screenings or vaccination)?	Use	Organization
D19	to find information on digital health services (like telemedicine, virtual consultation, access to your health records online) provided in your province?	to find information on digital health services provided in your province (such as telemedicine, virtual consultation, access to your health records online)?	Find	Organization
D20	to use digital health services (like telemedicine, virtual consultation)?	to use digital health services (such as telemedicine, virtual consultation)?	Use	Organization
D21	to find information about accessing emergency services?	to find information about accessing emergency services?	Find	Organization
D22	to find out if a doctor is affiliated with a specific private health insurance provider you may have?	to find out if your health insurance covers visits to certain health providers?	Assess	System
D23	to apply for provincial health plans or private health insurance?	to apply for provincial health plans or private health insurance?	Use	Organization
D24	to be confident assessing the information on healthcare coverage details from different sources?	to assess the information on healthcare coverage details from different sources?	Assess	System
D25	to use your insurance benefits for different health services?	to use your health insurance coverage for different health services?	Use	Organization

**Table 3:** Item reduction.

Item	How Easy Is It for You…	Loading Factors 25 Items	Loading Factors 20 Retained Items	Communality Value	Health Literacy Dimension	Navigational Health System Level
D1	to understand information on how the healthcare system works?	0.42	0.43	0.18	Understand	System
D2	to understand what different healthcare providers do?	0.44	0.45	0.20	Understand	System
D3	to know how long you’ll wait for a health appointment?	0.44	0.45	0.20	Understand	Organization
D4	to find out which healthcare services you can get, either through your provincial health plan or private insurance?	0.45	0.44	0.19	Find	Organization
D5	to figure out how much your health insurance covers for a specific service?	0.46	0.45	0.20	Assess	System
D6	to find a family doctor or primary healthcare provider near you?	0.37				
D7	to understand how to get an appointment with a particular health service?	0.42	0.41	0.17	Understand	Organization
D8	to book an appointment with a healthcare provider?	0.38				
D9	to find the right person to talk about your health concern within a healthcare institution?	0.47	0.46	0.21	Find	Organization
D10	to understand your rights as a patient or user of the healthcare system?	0.44	0.43	0.19	Understand	System
D11	to assess the quality of a particular health service?	0.42	0.44	0.20	Assess	Organization
D12	to assess whether a health service will meet your expectations and needs in case of a health problem?	0.42	0.43	0.18	Assess	System
D13	to find support options that help you navigate the healthcare system?	0.48	0.49	0.24	Find	Organization
D14	to decide a particular health service (such as choose from different hospitals)?	0.49	0.50	0.25	Use	Organization
D15	to stand up for yourself if your healthcare does not meet your needs?	0.48	0.48	0.23	Use	Interaction
D16	to talk with your healthcare providers and make decision together?	0.44	0.42	0.18	Use	Interaction
D17	to find information on preventive health services (such as screenings or vaccinations)?	0.41	0.40	0.16	Find	Organization
D18	to access preventive health services (such as screenings or vaccination)?	0.31				
D19	to find information on digital health services provided in your province (such as telemedicine, virtual consultation, access to your health records online)?	0.51	0.51	0.26	Find	Organization
D20	to use digital health services (such as telemedicine, virtual consultation)?	0.34				
D21	to find information about accessing emergency services?	0.46	0.44	0.20	Find	Organization
D22	to find out if your health insurance covers visits to certain health providers	0.48	0.48	0.23	Assess	System
D23	to apply for provincial health plans or private health insurance?	0.39				
D24	to access the information on healthcare coverage details from different sources?	0.46	0.47	0.22	Assess	System
D25	to use your health insurance coverage for different health services?	0.45	0.45	0.20	Use	Organization

**Table 4:** Fit indices for one-factor CFA model in each fold.

Indices	One-Factor Model		
	Fold = 1	Fold = 2	Fold = 3
n	227	227	227
CFI	0.97	0.96	0.96
TLI	0.97	0.96	0.95
SRMR	0.06	0.07	0.07
RMSEA_(90%CI)_	0.04 (0.03–0.06)	0.05 (0.04–0.06)	0.05 (0.03–0.06)
$\chi^{2}$	244.79	260.97	248.38
*df*	170	170	170
$\chi^{2}$/*df*	1.40	1.54	1.46

**Table 5:** Association between health system literacy score and sociodemographic characteristics.

		Dichotomous Score		Polytomous Score	
Group	N	Mean Score (SD)	*p*-Value	Mean Score (SD)	*p*-Value
Gender (A3)			**0.01 ^ab^**		**<0.01 ^ab^**
a. Men	305	32.79 (17.87)		48.26 (11.31)	
b. Women	375	28.41 (22.21)		45.06 (15.69)	
Effect size Hedge’s (a vs. b)		0.22 (0.06–0.37)		0.230 (0.08−0.38)	
Age group (A4a)			**<0.01 ^abc, ¥ab,¥ac,¥bc^**		**<0.01 ^abc,ab,ac,bc^**
a. Under 50	248	36.78 (19.34)		49.70 (12.40)	
b. 50 to 64	341	27.97 (20.48)		45.91 (13.98)	
c. 65 or older	92	21.20 (18.65)		40.11 (15.54)	
Effect size Hedge’s (a vs. b)		0.44 (0.27–0.61)		0.28 (0.12–0.45)	
Effect size Hedge’s (a vs. c)		0.76 (0.51–1.01)		0.72 (0.47–0.97)	
Effect size Hedge’s (b vs. c)		0.29 (0.05–0.52)		0.41 (0.17–0.64)	
Highest level of education (A7a)			**<0.01 ^abc,§ab^**		**<0.01 ^abc,ab,bc^**
a. College/university	309	27.67 (21.55)		44.19 (15.19)	
b. Postgraduate (master/Ph.D.)	356	33.24 (18.96)		48.96 (12.01)	
c. Other (high school, others)	16	19.33 (22.19)		36.00 (18.58)	
Effect size Hedge’s (a vs. b)		−0.28 (−0.43–−0.12)		−0.35 (−0.51–−0.20)	
Effect size Hedge’s (a vs. c)		0.39 (−0.13–0.91) ^‡^		0.53 (0.01–1.05)	
Effect size Hedge’s (b vs. c)		0.73 (0.21–1.25)		1.05 (0.53–1.57)	
Income level (A12a)			**<0.01 ^abc,¥ab,¥ac^**		**<0.01 ^abc,ab,ac,bc^**
a. <CAD 60,000	92	18.59 (23.24)		38.01 (18.69)	
b. CAD 60,000 to CAD 89,999	231	35.20 (18.01)		50.83 (10.57)	
c. ≥CAD 90,000	305	33.07 (18.92)		47.83 (11.84)	
Effect size Hedge’s (a vs. b)		−0.84 (−1.10–−0.59)		−0.96 (−1.21–−0.70)	
Effect size Hedge’s (a vs. c)		−0.72 (−0.96–−0.48)		−0.71 (−0.95–−0.48)	
Effect size Hedge’s (b vs. c)		0.12 (−0.06–0.29) ^‡^		0.27 (0.09–0.44)	
Length of stay (LOS) in Canada (A6)			0.53		0.53
a. LOS < 5 years	56	33.30 (22.91)		46.33 (16.47)	
b. 5 years ≤ LOS < 10 years	102	30.39 (19.43)		45.11 (14.14)	
c. LOS ≥ 10 years	522	30.07 (20.42)		46.80 (13.67)	
Effect size Hedge’s (a vs. b)		0.14 (−0.19–0.47) ^‡^		0.08 (−0.25–0.41) ^‡^	
Effect size Hedge’s (a vs. c)		0.16 (−0.12–0.43) ^‡^		−0.03 (−0.31–0.24) ^‡^	
Effect size Hedge’s (b vs. c)		0.02 (−0.20–0.23) ^‡^		−0.12 (−0.34–0.09) ^‡^	
Marital status (A8a)			0.051		0.07
a. Married	588	31.01 (20.28)		46.96 (13.57)	
b. Others	93	26.53 (21.35)		43.67 (16.10)	
Effect size Hedge’s (a vs. b)		0.22 (0.00–0.44) ^‡^		0.24 (0.02–0.46) ^‡^	
Current employment status (A9a)			**0.01 ^abc,§ac^**		0.06
a. Employed	307	32.11 (20.03)		47.77 (12.89)	
b. Self-employed	120	32.15 (23.99)		46.46 (15.44)	
c. Others	254	27.51 (18.91)		45.00 (14.41)	
Effect size Hedge’s (a vs. b)		0.00 (−0.21–0.21) ^‡^		0.09 (−0.12–0.31)	
Effect size Hedge’s (a vs. c)		0.24 (0.07–0.40)		0.20 (0.04–0.37)	
Effect size Hedge’s (b vs. c)		0.22 (0.01–0.44)		0.10 (−0.12–0.32)	
Self-rated health (B1a)			**0.01 ^abc,¥ab^**		**0.01 ^abc,ab,ac^**
a. Excellent to good	271	33.16 (21.21)		48.44 (13.57)	
b. Good	243	27.53 (20.03)		45.51 (14.03)	
c. Fair to Poor	164	30.16 (19.35)		44.92 (14.18)	
Effect size Hedge’s (a vs. b)		0.27 (0.10–0.45)		0.21 (0.04–0.39)	
Effect size Hedge’s (a vs. c)		0.15 (−0.05–0.34) ^‡^		0.26 (0.06–0.45)	
Effect size Hedge’s (b vs. c)		−0.13 (−0.332–0.07) ^‡^		0.04 (−0.16–0.24)	
Number of non-cancer chronic comorbidities (B3a)			**<0.01 ^abcde,§ac,§bc,§bd^**		**0.03 ^abcde^**
a. None	268	29.06 (23.76)		46.87 (15.78)	
b. 1	150	27.44 (20.22)		44.93 (15.19)	
c. 2	177	35.14 (13.87)		48.25 (8.95)	
d. >2	60	35.17 (14.64)		48.17 (9.63)	
e. Don’t know	19	17.11 (26.84)		36.84 (19.69)	
Effect size Hedge’s (a vs. b)		0.07 (−0.13–0.27) ^‡^		0.13 (−0.08–0.33) ^‡^	
Effect size Hedge’s (a vs. c)		−0.30 (−0.49–−0.11)		−0.10 (−0.29–0.09) ^‡^	
Effect size Hedge’s (a vs. d)		−0.27 (−0.55–0.01) ^‡^		−0.09 (−0.37–0.19) ^‡^	
Effect size Hedge’s (a vs. e)		0.50 (0.03–0.97)		0.62 (0.15–1.09)	
Effect size Hedge’s (b vs. c)		−0.45 (−0.67–−0.23)		−0.27 (−0.49–−0.05)	
Effect size Hedge’s (b vs. d)		−0.41 (−0.71–−0.11)		−0.23 (−0.53–0.07) ^‡^	
Effect size Hedge’s (b vs. e)		0.49 (0.01–0.97)		0.51 (0.03–0.99)	
Effect size Hedge’s (c vs. d)		0.00 (−0.30–0.29) ^‡^		0.01 (−0.29–0.30) ^‡^	
Effect size Hedge’s (c vs. e)		1.16 (0.67–1.64)		1.09 (0.60–1.58)	
Effect size Hedge’s (d vs. e)		0.98 (0.44–1.52)		0.88 (0.35–1.42)	
Diagnosed with cancer (B4b)			**0.04 ^ab^**		0.36
a. Yes	551	29.91 (21.31)		46.42 (14.47)	
b. No	125	33.44 (15.82)		47.51 (11.13)	
Effect size Hedge’s (a vs. b)		−0.17 (−0.37–0.02) ^‡^		−0.08 (−0.27–0.12) ^‡^	
Having a general practitioner/family physician (C3)			0.19		**0.02 ^ab^**
a. Yes	626	30.76 (19.98)		47.03 (13.23)	
b. No	54	26.02 (25.33)		40.26 (19.86)	
Effect size Hedge’s (a vs. b)		0.23 (−0.05–0.51) ^‡^		0.49 (0.21–0.77)	
Need assistance to see an HCP (C5)			**<0.01 ^ab^**		0.99
a. Yes	302	33.86 (15.25)		46.52 (11.88)	
b. No	375	27.67 (23.54)		46.53 (15.52)	
Effect size Hedge’s (a vs. b)		0.31 (0.15–0.46)		0.00 (−0.15–0.15) ^‡^	

abc, abcde: *p*-value from ANOVA test indicating unequal means across all groups. ab, ac, bc, bd: *p*-value from *t* test or post hoc test indicating different means between two groups. **^¥^** The post hoc test using Tukey HSD for homogeneous variance with *p*-value < 0.05 indicates a significant difference. **^§^** The post hoc test using Games–Howell for heterogenous variance with *p*-value < 0.05 indicates a significant difference. Means highlighted in bold are significant at *p* < 0.05 for difference mean using *t*-test or ANOVA; ES (effect size) calculated using Hedges’ g for standardized difference in means with 0.2 ≤ Hedges’ g value < 0.5 indicating small ES, 0.5 ≤ Hedges’ g < 0.8 indicating medium ES, and Hedges’ g > 0.8 indicating large ES and all significant ES highlighted in bold. ^‡^ An effect size may be negligible.

## Data Availability

Data used in this study are available from the corresponding author to provide the necessary context, reduce the risk of misinterpretation, and ensure that the data are used for appropriate scholarly purposes.

## Supplementary Materials

The following supporting information can be downloaded at https://www.mdpi.com/article/10.3390/healthcare13192410/s1. Supplementary S1. COSMIN Reporting Checklist for studies on measurement properties. Supplementary S2. Original HLS19—NAV instrument. Supplementary S3. Summary of respondents for each item in HSL-CAN scale and HLS-SF12. Supplementary S4. Psychometric evaluation for HLS-SF12 scale. Supplementary S5. Check multicollinearity. Supplementary S6. A comparison of models from EFA and theoretical frameworks. Supplementary S7. Confirmatory factor analysis for three-factors: health system literacy, health care, and prevention and health promotion. Supplementary S8. Difference in means of health system literacy scores among sociodemographic groups using t-test, one-way ANOVA, pair-wise post-hoc tests.
