# Supplementary material for: Developing a Health System Literacy Measure for Chinese Immigrants in Canada: Adapting the HLS19–NAV Scale
Source: Healthcare (Basel). 2025 Sep 24;13(19):2410. doi: 10.3390/healthcare13192410 (PMC12523927; doi:10.3390/healthcare13192410)
Supplement: Supplementary file 1 [file healthcare-13-02410-s001.zip › healthcare-3860240-supplementary.pdf]

Supplementary S1. COSMIN Reporting Checklist for studies on measurement properties

Table S1.1. General Reporting recommendations relevant for all studies on measurement properties

Item name	Item description	Page
**Report section: Title**		
Title	Identify the report as a study of one or more measurement properties of a specific PROM to measure a specified construct in a specified population.	p1
Objectives	Provide the specific objective(s) of the research, specifying (1) the name (and version, if relevant), and construct(s) of the PROM, (2) the measurement properties being evaluated, and (3) relevant study characteristics.	p1
Design	Specify (details of the) study design used to evaluate the measurement properties.	p1
Methods	Specify the methods for evaluating each measurement property.	p1
Results	Provide the main results for all measurement properties evaluated.	p1
Discussion/ Conclusions	Provide a brief statement of the implications of the findings in the context of existing evidence on the PROM.	p1
**Report section: Introduction**		
PROM	Specify the name and, if relevant, the version, and construct(s) of the PROM.	p2
Target population & context of use	Specify the target population and context of use that the PROM was designed for.	p2
State of knowledge & Rationale	Provide a description of the current scientific knowledge (what is known and not known) regarding the measurement properties of the PROM. Explain why the new study is necessary. Provide citations for the original development paper(s).	p2
Objectives	Provide the specific objective(s) of the research, specifying (1) the name (and version, if relevant) of the PROM, (2) the measurement properties being evaluated, and (3) relevant study sample characteristics.	p2
**Report section: General Methods**		
Study design	Specify (details of the) study design used to evaluate the measurement properties.	p2-3
Participants	Specify how the study participants were selected. Specify the inclusion and exclusion criteria	p2-5
PROM details	Provide details about the original version of the PROM as well as of the PROM version being studied, specify the conceptual framework (reflective/formative model), details on the structure (the number of items and subscales), the language, response scale, recall period, direction of scoring, and scoring algorithm of the PROM. Specify how the PROM was administered (e.g., in what setting, mode of administration (e.g. paper, electronic) what instructions were given), including the country in which it is administered	p5
Additional data collection	Describe why and how other data was collected (e.g., construct and measurement properties of the comparator instruments, characteristics of groups being compared, and rationale for choosing groups), including mode of administration (e.g., paper, electronic).	p3

Item name	Item description	Page
Time points procedures	Provide all time points of all measurements.	p2
Justification for sample size	Provide a rationale for the sample size for all measurement properties analyses (including subgroups).	p2-3
Statistical analyses	Describe the statistical analyses corresponding to all objectives (see measurement properties specific boxes). Describe the criteria for good measurement properties. Name the statistical package used and the version.	p6-8
Missing data	Describe approaches for dealing with missing data.	p8
Unplanned analysis	Specify analyses that were unplanned and their rationale.	n/a
**Report section: General results**		
Participant characteristics	Provide study participants' characteristics, specified per subgroup if applicable.	p2-3
Sample size	Provide the total number of participants included in the study and the sample size for each analysis.	p2-3
Missing data	Provide amount of (proportion or count) and reasons for missing data for each analysis for the PROM, and for any analyses of other outcome measurement instruments.	p4-5, Suppl 3
Results	Describe the results corresponding to all objectives (see measurement properties specific boxes).	p8-15
**Report section: Discussion/conclusions**		
Measurement property evidence	Provide the main findings and if each measurement property is sufficient or insufficient and why.	p15-17
Practical relevance	Discuss the practical relevance of the findings in terms of recommendations for (not) using the PROM.	p17-18
Strengths and limitations	Discuss strengths and limitations of each study. For example, discuss if there were any potential biases in the study that could have impacted the results.	p17-18
Generalizability	Discuss generalizability of the results. For example, discuss whether the results could be generalized to other populations given the sample studied.	n/a
Instrument changes	Discuss what modifications are needed to the existing PROM.	p18
Future research	Describe new research questions or hypotheses generated from these findings and provide/ describe the research needed to answer those questions.	p18
Conclusions	Provide the overall conclusions for the use of the PROM.	p18
**Report section: Other information**		
Conflict of interest	State any conflict of interest you may have related to the PROM. This may include any involvement in the development of the PROM or any commercial funding or profit.	p19

Table S1.2. Revised items for reporting studies on measurement properties of Patient-Reported Outcome Measures (PROMs): Specific Reporting recommendations for studies

**Specific Reporting recommendations for studies on Content Validity**		
**Content validity: Methods**		
Relevance	Specify if and how patients and/or professionals were asked whether the instructions, each of the items, response options, and the recall period were relevant for the construct(s), population, and context of use.	p6
Comprehensiveness	Specify whether and how patients and/or professionals were asked whether all key concepts are included in the PROM.	p6
Comprehensibility	Specify whether and how the comprehensibility of the PROM instructions, items, response options, and recall period was evaluated by patients and/or professionals.	p6
**Content validity: Results**		
Relevance	Specify if the instructions, all items, response options, and recall period were considered relevant, by patients and/or professionals, to the construct, population, and context of use.	p10, p15
Comprehensiveness	Specify whether patients and/or professionals considered all key concepts to be included in the PROM.	p10, p15
Comprehensibility	Specify whether patients understood the PROM instructions, items, response options, and recall period as intended and/or whether professionals considered the instructions, items, response options, were appropriately worded.	p10, p15
**Specific Reporting recommendations for studies on Structural Validity**		
**Structural validity: Methods**		
Rationale for approach	Provide a rationale for the approach (e.g., factor analysis, Item Response Theory (IRT)/Rasch analysis) used.	p6-7
Statistical analyses	Exploratory (EFA) or confirmatory factor analyses (CFA)<br>Describe the tested model (e.g., number of factors, which items included in which factor), method of estimation, type of correlation matrix, and methods and criteria for good model fit.<br><br>IRT/Rasch analysis<br>Describe the type of IRT/Rasch model, method of estimation, methods for checking assumptions (e.g., dimensionality, local independence, monotonicity), methods and criteria for good item parameters and model fit. Indicator software and version used.<br><br>Other approaches<br>Provide details of the methods if other approaches were used.	p6-7
**Structural validity: Results**		
Statistical analyses	EFA or CFA<br>For EFA: provide all factor loadings, eigenvalues and % variance explained of the model reflecting the original PROM structure and best-fitting model if applicable.<br><br>IRT/Rasch analyses	p10-12

	Provide item/model fit results, all item parameters, and figures if appropriate (e.g., item characteristic curves, person-item mapping, item and/or test information functions).<br>Other approaches<br>Provide relevant results for other approaches used.	
**Specific Reporting recommendations for studies on Internal Consistency**		
**Internal Consistency: Methods**		
Statistical analyses	Provide evidence for the unidimensionality of the PROM (subscales) and provide evidence of lack of local item dependence. Describe statistical methods used to calculate internal consistency.	p7
**Internal Consistency: Results**		
Statistical analyses	Provide internal consistency results for each unidimensional scale or subscale separately.	p12
**Specific Reporting recommendations for studies on Hypotheses Testing for Construct Validity**		
**Hypotheses Testing for Construct Validity: Methods**		
Hypotheses	State hypotheses and provide the rationale for each hypothesis.	p7-8
Statistical analyses	Specify all statistical methods used to test the hypotheses.	p7-8
**Hypotheses Testing for Construct Validity: Results**		
Statistical analyses	Provide all results and specify if each result is in accordance with its hypothesis.	p13-15

Supplementary S2. Original HLS_{19} – NAV instrument

Table S2. The HLS_{19} – NAV instrument

How easy would you say it is…?	
Item 1	to understand information on how the health care system works?
Item 2	to judge which type of health service you need in case of a health problem?
Item 3	to judge to what extent your health insurance covers a particular health service?
Item 4	to understand information on ongoing health care reforms that might affect your health care?
Item 5	to find out about your rights as a patient or user of the health care system?
Item 6	to decide for a particular health service?
Item 7	to find information on the quality of a particular health service?
Item 8	to judge if a particular health service will meet your expectations and wishes on health care?
Item 9	to understand how to get an appointment with a particular health service?
Item 10	to find out about support options that may help you to orientate yourself in the health care system?
Item 11	to locate the right contact person for your concern within a health care institution?
Item 12	to stand up for yourself if your health care does not meet your needs?

Supplementary S3. Summary of respondents for each item in the HSL-CAN scale and the HLS-SF12

Table S3. Summary of respondents on the HSL-CAN scale (D1-D25) and the HLS-SF12 (D34-D45)

Item	Extremely difficult n (%)	Somewhat difficult n (%)	Neither easy nor difficult n (%)	Somewhat easy n (%)	Extremely easy n (%)	Missing n (%)
D1	69 (10.1)	197 (28.9)	215 (31.6)	144 (21.1)	56 (8.22)	0 (0.00)
D2	73 (10.7)	188 (27.6)	190 (27.9)	159 (23.3)	71 (10.4)	0 (0.00)
D3	94 (13.8)	189 (27.8)	203 (29.8)	129 (18.9)	66 (9.69)	0 (0.00)
D4	87 (12.8)	179 (26.3)	200 (29.4)	145 (21.3)	68 (9.99)	2 (0.30)
D5	92 (13.5)	187 (27.5)	186 (27.3)	151 (22.2)	64 (9.40)	1 (0.15)
D6	103 (15.1)	154 (22.6)	179 (26.3)	174 (25.6)	71 (10.4)	0 (0.00)
D7	100 (14.7)	176 (25.8)	184 (27.0)	154 (22.6)	67 (9.84)	0 (0.00)
D8	74 (10.9)	170 (25.0)	205 (30.1)	144 (21.1)	88 (12.9)	0 (0.00)
D9	97 (14.2)	197 (28.9)	185 (27.2)	132 (19.4)	70 (10.3)	0 (0.00)
D10	82 (12.0)	183 (26.9)	206 (30.2)	140 (20.6)	69 (10.1)	1 (0.15)
D11	87 (12.8)	166 (24.4)	198 (29.1)	159 (23.3)	71 (10.4)	0 (0.00)
D12	83 (12.2)	162 (23.8)	213 (31.3)	149 (21.9)	70 (10.3)	4 (0.59)
D13	85 (12.5)	174 (25.6)	214 (31.4)	152 (22.3)	55 (8.08)	1 (0.15)
D14	105 (15.4)	191 (28.0)	186 (27.3)	129 (18.9)	68 (9.99)	2 (0.29)
D15	127 (18.6)	208 (30.5)	164 (24.1)	113 (16.6)	68 (9.99)	1 (0.15)
D16	104 (15.3)	168 (24.7)	199 (29.2)	155 (22.8)	53 (7.78)	2 (0.29)
D17	83 (12.2)	155 (22.8)	215 (31.6)	158 (23.2)	69 (10.1)	1 (0.15)
D18	77 (11.3)	156 (22.9)	198 (29.1)	186 (27.3)	63 (9.25)	1 (0.15)
D19	90 (13.2)	181 (26.6)	214 (31.4)	124 (18.2)	71 (10.4)	1 (0.15)
D20	78 (11.5)	178 (26.1)	227 (33.3)	139 (20.4)	56 (8.22)	3 (0.44)
D21	87 (12.8)	178 (26.1)	212 (31.1)	140 (20.6)	61 (8.96)	3 (0.44)
D22	94 (13.8)	191 (28.0)	202 (29.7)	134 (19.7)	56 (8.22)	4 (0.59)
D23	92 (13.5)	170 (25.0)	210 (30.8)	135 (19.8)	67 (9.84)	7 (1.03)
D24	115 (16.9)	200 (29.4)	173 (25.4)	119 (17.5)	72 (10.6)	2 (0.29)
D25	96 (14.1)	181 (26.6)	196 (28.8)	135 (19.8)	63 (9.25)	10 (1.47)
D34	82 (12.0)	168 (24.7)	208 (30.5)	149 (21.9)	71 (10.4)	3 (0.44)
D35	84 (12.3)	141 (20.7)	185 (27.2)	194 (28.5)	73 (10.7)	4 (0.59)
D36	102 (15.0)	181 (26.6)	185 (27.2)	114 (16.7)	96 (14.1)	3 (0.44)
D37	69 (10.1)	132 (19.4)	177 (26.0)	201 (29.5)	98 (14.4)	4 (0.59)
D38	81 (11.9)	150 (22.0)	225 (33.0)	157 (23.1)	64 (9.40)	4 (0.59)
D39	61 (8.96)	97 (14.2)	155 (22.8)	219 (32.2)	143 (21.0)	6 (0.88)
D40	86 (12.6)	149 (21.9)	194 (28.5)	163 (23.9)	83 (12.2)	6 (0.88)
D41	64 (9.40)	127 (18.6)	204 (30.0)	205 (30.1)	76 (11.2)	5 (0.73)
D42	61 (8.96)	112 (16.4)	191 (28.0)	204 (30.0)	108 (15.9)	5 (0.73)
D43	70 (10.3)	118 (17.3)	204 (30.0)	199 (29.2)	87 (12.8)	3 (0.44)
D44	50 (7.34)	99 (14.5)	160 (23.5)	227 (33.3)	142 (20.9)	3 (0.44)
D45	41 (6.02)	126 (18.5)	204 (30.0)	195 (28.6)	111 (16.3)	4 (0.59)

Supplementary S4. Psychometric evaluation for the HLS-SF12 scale

Table S4.1. The HLS-SF12 scale

How easy would you say it is to	
D34	find information on treatments of illnesses that concern you?
D35	understand the leaflets that come with your medicine?
D36	assess the advantages and disadvantages of different treatment options?
D37	call ambulance in an emergency situation?
D38	find information on managing mental health problems like stress and depression?
D39	understand why you need health screenings (such as breast cancer, blood sugar test, blood pressure)
D40	judge which vaccinations you may need?
D41	decide how you can protect yourself from illness based on advice from family and friends?
D42	find out about activities (such as meditation, exercise, walking, Pilates) that are good for your mental well-being?
D43	understand information in the media (such as Internet, newspaper, magazines) on how to get healthier?
D44	judge which everyday behavior (such as drinking and eating habits, exercises) is related to your health?
D45	join a sport club or exercise class if you want to?

A. Exploratory factor analysis

The overall Kaiser-Meyer-Olkin values of 0.75 and Bartlett test of Sphericity with p-value < 0.05. The parallel analysis with weighted least square (wls) indicated the number of factors is two. The Scree plot showed two factors.

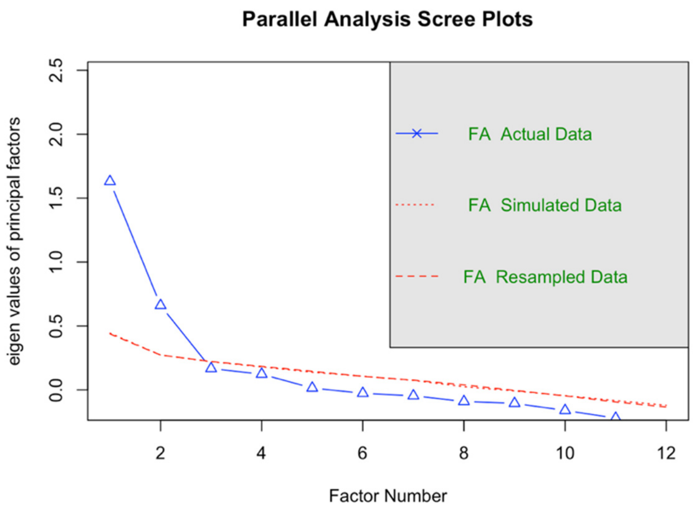

Figure S4.1 Parallel analysis and scree plot for exploratory factor analysis of HLS-SF12 scale.

Table S4.2. Item factor loadings of the HLS-SF12

Item	How easy can you	Factor 1	Factor 2
D34	understand information on how the healthcare system works?	0.09	0.43
**D35**	understand the leaflets that come with your medicine?	0.17	0.45
D36	assess the advantages and disadvantages of different treatment options?	- 0.14	0.63
D37	call ambulance in an emergency situation?	0.25	0.18
D38	find information on managing mental health problems like stress and depression?	0.12	0.39
D39	understand why you need health screenings (such as breast cancer, blood sugar test, blood pressure)	0.49	0.06
D40	judge which vaccinations you may need?	0.26	0.22
D41	decide how you can protect yourself from illness based on advice from family and friends?	0.29	0.14
D42	find out about activities (such as meditation, exercise, walking, Pilates) that are good for your mental well-being?	0.42	0.08
D43	understand information in the media (such as Internet, newspaper, magazines) on how to get healthier?	0.51	0.09
D44	judge which everyday behavior (such as drinking and eating habits, exercises) is related to your health?	0.55	0.00
D45	join a sport club or exercise class if you want to?	0.48	0.04

B. Confirmatory Factor Analysis and internal consistency

From the confirmatory Factor Analysis, fit indices for HLS-SF12 with two factors: CFI=0.946, TLI=0.933, RMSEA=0.035, SRMR=0.046, χ^2/df ratio of 1.81 (96.146/53) indicate a goodness of fit model. For internal consistency test, Cronbach's alpha coefficient of 0.53 for factor 1 (D34, D35, D36, D38) and 0.63 for factor 2 (D37, D39, D40, D41, D42, D43, D44, D45).

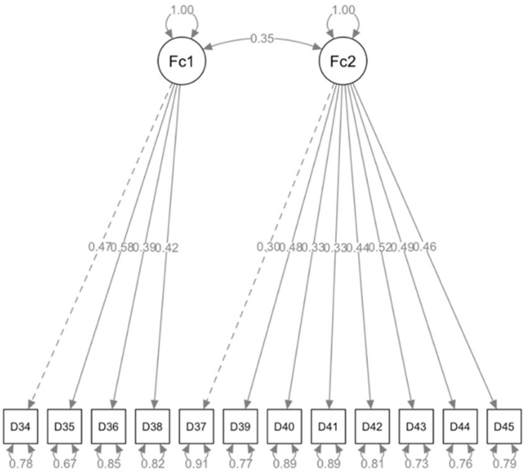

Figure S4.2. Path diagram of the confirmatory factor analysis

Supplementary S5. Check multicollinearity

Table S5.1. Correlation coefficient

Rows kept after list-wise deletion: 669 / 681

	D1	D2	D3	D4	D5	D7	D9	D10	D11	D12	D13	D14	D15	D16	D19	D21	D22	D24	D25
D1	1.00	0.28	0.23	0.18	0.25	0.17	0.11	0.12	0.22	0.14	0.14	0.16	0.15	0.19	0.16	0.16	0.19	0.24	0.17
D2	0.28	1.00	0.24	0.21	0.21	0.14	0.21	0.20	0.18	0.15	0.16	0.17	0.19	0.21	0.20	0.17	0.17	0.13	0.22
D3	0.23	0.24	1.00	0.19	0.16	0.23	0.18	0.19	0.21	0.11	0.24	0.18	0.22	0.14	0.24	0.19	0.18	0.15	0.15
D4	0.18	0.21	0.19	1.00	0.20	0.19	0.13	0.16	0.21	0.18	0.24	0.13	0.18	0.14	0.19	0.21	0.21	0.16	0.22
D5	0.25	0.21	0.16	0.20	1.00	0.17	0.13	0.16	0.19	0.23	0.20	0.19	0.14	0.15	0.18	0.22	0.18	0.21	0.20
D7	0.17	0.14	0.23	0.19	0.17	1.00	0.19	0.20	0.15	0.17	0.22	0.22	0.13	0.17	0.18	0.12	0.20	0.15	0.07
D9	0.11	0.21	0.18	0.13	0.13	0.19	1.00	0.20	0.19	0.21	0.23	0.19	0.19	0.20	0.21	0.21	0.26	0.21	0.19
D10	0.12	0.20	0.19	0.16	0.16	0.20	0.20	1.00	0.22	0.13	0.27	0.15	0.20	0.18	0.22	0.14	0.17	0.15	0.18
D11	0.22	0.18	0.21	0.21	0.19	0.15	0.19	0.22	1.00	0.27	0.19	0.20	0.18	0.15	0.16	0.15	0.18	0.16	0.20
D12	0.14	0.15	0.11	0.18	0.23	0.17	0.21	0.13	0.27	1.00	0.19	0.26	0.19	0.19	0.19	0.10	0.17	0.19	0.16
D13	0.14	0.16	0.24	0.24	0.20	0.22	0.23	0.27	0.19	0.19	1.00	0.23	0.21	0.16	0.21	0.19	0.18	0.17	0.25
D14	0.16	0.17	0.18	0.13	0.19	0.22	0.19	0.15	0.20	0.26	0.23	1.00	0.24	0.21	0.20	0.21	0.26	0.21	0.22
D15	0.15	0.19	0.22	0.18	0.14	0.13	0.19	0.20	0.18	0.19	0.21	0.24	1.00	0.27	0.20	0.19	0.18	0.22	0.15
D16	0.19	0.21	0.14	0.14	0.15	0.17	0.20	0.18	0.15	0.19	0.16	0.21	0.27	1.00	0.16	0.17	0.15	0.18	0.17
D19	0.16	0.20	0.24	0.19	0.18	0.18	0.21	0.22	0.16	0.19	0.21	0.20	0.20	0.16	1.00	0.24	0.24	0.26	0.22
D21	0.16	0.17	0.19	0.21	0.22	0.12	0.21	0.14	0.15	0.10	0.19	0.21	0.19	0.17	0.24	1.00	0.19	0.20	0.18
D22	0.19	0.17	0.18	0.21	0.18	0.20	0.26	0.17	0.18	0.17	0.18	0.26	0.18	0.15	0.24	0.19	1.00	0.28	0.16
D24	0.24	0.13	0.15	0.16	0.21	0.15	0.21	0.15	0.16	0.19	0.17	0.21	0.22	0.18	0.26	0.20	0.28	1.00	0.18
D25	0.17	0.22	0.15	0.22	0.20	0.07	0.19	0.18	0.20	0.16	0.25	0.22	0.15	0.17	0.22	0.18	0.16	0.18	1.00

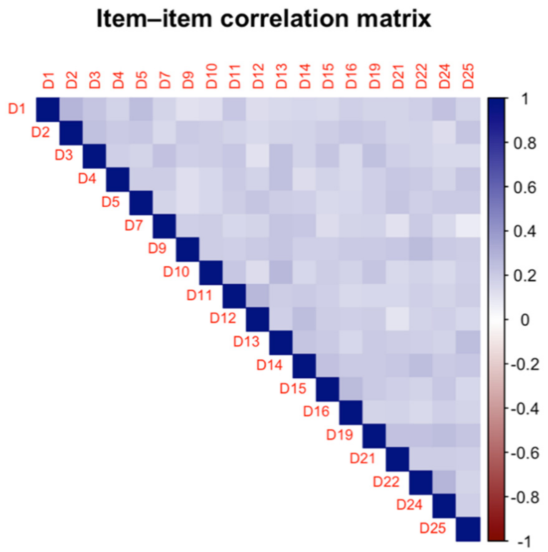

Figure S5. Item-item correlation matrix

Table S5.2 Variance Inflation Factor value

Variable	VIF
D13	1.26
D3	1.24
D14	1.24
D22	1.24
D1	1.23
D2	1.23
D19	1.23
D24	1.23
D9	1.22
D12	1.22
D25	1.22
D5	1.21
D11	1.21
D15	1.21
D4	1.20
D7	1.19
D10	1.19
D21	1.19
D16	1.17

Supplementary S6. A comparison of models from EFA and theoretical frameworks.

Table S6. Goodness-of-fit indices for the models

Indices	One-factor	Two-factor	Four-factor	
		**Correlated**	**Correlated**	**Hierarchy**
**CFA**				
**Fit indices**				
n	669	669	669	669
CFI	0.99	0.99	0.99	0.99
TLI	0.99	0.99	0.99	0.99
SRMR	0.04	0.04	0.04	0.04
RMSEA (90%CI)	0.03 (0.02–0.03)	0.03 (0.02-0.03)	0.02 (0.02 –0.03)	0.02 (0.02 – 0.03)
χ^2	250.28	249.03	229.18	229.40
df	170	169	164	166
χ^2/df	1.75	1.76	1.63	1.61
**Inter-factor correlation**				
F1 ~ F2	-	0.97	0.88	-
F1 ~ F3	-	-	0.84	-
F1 ~ F4	-	-	0.83	-
F2 ~ F3	-	-	0.92	-
F2 ~ F4	-	-	0.94	-
F3 ~ F4	-	-	0.89	-
G ~ F1	-	-	-	0.89
G ~ F2	-	-	-	0.99
G ~ F3	-	-	-	0.94
G ~ F4	-	-	-	0.94
**Reliability**				
**Cronbach's alpha**				
Overall score	0.82	0.82	0.82	0.82
F1	-	0.62	0.56	0.56
F2	-	0.75	0.60	0.60
F3	-	-	0.57	0.57
F4	-	-	0.51	0.51
**Composite reliability**				
Overall score	0.82	0.82	0.82	0.82
F1	-	0.63	0.57	0.57
F2	-	0.75	0.59	0.59
F3	-	-	0.57	0.57
F4	-	-	0.52	0.52

Note:
G: health system literacy
Four-factor model: F1- Understand; F2- Find; F3- Assess; F4- Apply
Two-factor model: F1- System level; F- Organizational & interaction level

Supplementary S7. Confirmatory factor analysis for three-factors: health system literacy, health care, and prevention and health promotion

Table S7. Correlation coefficients between the HSL-CAN scale and the subscales of the HLS-SF12

Measures/ subscales	Coefficients	Std. Err	z-value	p-value
New scale ~~				
Health care subscale	0.79	0.04	17.93	0.000
Prevention and health promotion subscale	0.21	0.06	3.10	0.002
Health care subscale ~~				
Prevention and health promotion subscale	0.32	0.07	4.93	0.000

Supplementary S8. Difference in means of health system literacy scores among sociodemographic groups using t-test, one-way ANOVA, pair-wise post-hoc tests

Table S8. Outputs from t-test, one-way ANOVA, and pair-wise post-hoc tests

		**Dichotomous score**			**Polytomous score**		
**Group**	**N**	**Mean score (SD)**	**F-value**	**p-value**	**Mean score (SD)**	**F-value**	**p-value**
**Gender (A3)**				**0.005**			**0.002**
a. Men	305	32.79 (17.87)			48.26 (11.31)		
b. Women	375	28.41 (22.21)			45.06 (15.69)		
Difference means (a vs. b)		4.38			3.20		
**Age group (A4a)**			**23.13**	**0.000**		17.08	**0.000**
a. Under 50	248	36.78 (19.34)			49.70 (12.40)		
b. 50 to 64	341	27.97 (20.48)			45.91 (13.98)		
c. 65 or older	92	21.20 (18.65)			40.11 (15.54)		
Difference means (a vs. b)		8.81		0.000‡	3.79		0.003‡
Difference means (a vs. c)		14.58		0.000‡	9.59		0.000‡
Difference means (b vs. c)		5.77		0.037‡	5.80		0.001‡
**Highest level of education (A7a)**			**8.05**	**0.001**		12.37	**0.000**
a. College/University	309	27.67 (21.55)			44.19 (15.19)		
b. Postgraduate (Master/PhD)	356	33.24 (18.96)			48.96 (12.01)		
c. Other (high school, others)	16	19.33 (22.19)			36.00 (18.58)		
Difference means (a vs. b)		-5.57		0.001¥	-4.77		0.000¥
Difference means (a vs. c)		8.33		0.355¥	8.19		0.245¥
Difference means (b vs. c)		13.90		0.074¥	12.96		0.044¥
**Income level (A12a)**			**25.80**	**0.000**		20.46	**0.000**
a. < \$60,000	92	18.59 (23.24)			38.01 (18.69)		
b. \$60,000 to \$89,999	231	35.20 (18.01)			50.83 (10.57)		
c. ≥ \$90,000	305	33.07 (18.92)			47.83 (11.84)		
Difference means (a vs. b)		16.61		0.000‡	-12.82		0.000¥
Difference means (a vs. c)		14.48		0.000‡	-9.82		0.000¥
Difference means (b vs. c)		-2.14		0.414‡	3.00		0.006¥
**Length of stay (LOS) in Canada (A6)**			0.63	0.533		0.63	0.533
a. LOS < 5 years	56	33.30 (22.91)			46.33 (16.47)		
b. 5 years ≤ LOS < 10 years	102	30.39 (19.43)			45.11 (14.14)		
c. LOS ≥ 10 years	522	30.07 (20.42)			46.80 (13.67)		
Difference means (a vs. b)		2.91	n/a		1.22		n/a

		Dichotomous score			Polytomous score		
**Group**	**N**	**Mean score (SD)**	**F-value**	**p-value**	**Mean score (SD)**	**F-value**	**p-value**
Difference means (a vs. c)		3.23	n/a		-0.47		n/a
Difference means (b vs. c)		0.32	n/a		-1.69		n/a
**Marital status (A8a)**				0.051			0.065
a. Married	588	31.01 (20.28)			46.96 (13.57)		
b. Others	93	26.53 (21.35)			43.67 (16.10)		
Difference means (a vs. b)		4.48			3.29		
**Current employment status (A9a)**				**0.014**		2.74	0.064
a. Employed	307	32.11 (20.03)			47.77 (12.89)		
b. Self-employed	120	32.15 (23.99)			46.46 (15.44)		
c. Others	254	27.51 (18.91)			45.00 (14.41)		
Difference means (a vs. b)		-0.04		1.000¥	1.29		n/a
Difference means (a vs. c)		4.59		0.015¥	2.77		n/a
Difference means (b vs. c)		4.63		0.155¥	1.49		n/a
**Self-rated health (B1a)**			**4.33**	**0.008**		**4.30**	**0.014**
a. Excellent to good	271	33.16 (21.21)			48.44 (13.57)		
b. Good	243	27.53 (20.03)			45.51 (14.03)		
c. Fair to Poor	164	30.16 (19.35)			44.92 (14.18)		
Difference means (a vs. b)		5.63		0.005‡	-2.92		0.046‡
Difference means (a vs. c)		3.00		0.297‡	-3.52		0.029‡
Difference means (b vs. c)		-2.63		0.411‡	-0.60		0.905‡
**Number of non-cancer chronic comorbidities (B3a)**			**7.21**	**0.000**		**2.86**	**0.027**
a. None	268	29.06 (23.76)			46.87 (15.78)		
b. 1	150	27.44 (20.22)			44.93 (15.19)		
c. 2	177	35.14 (13.87)			48.25 (8.95)		
d. > 2	60	35.17 (14.64)			48.17 (9.63)		
e. Don’t know	19	17.11 (26.84)			36.84 (19.69)		
Difference means (a vs. b)		1.62		0.948¥	1.95		0.730¥
Difference means (a vs. c)		-6.09		0.007¥	-1.37		0.770¥
Difference means (a vs. d)		-6.11		0.083¥	-1.29		0.920¥
Difference means (a vs. e)		11.95		0.355¥	10.03		0.230¥
Difference means (b vs. c)		-7.71		0.001¥	-3.32		0.130¥
Difference means (b vs. d)		-7.73		0.020¥	-3.24		0.350¥
Difference means (b vs. e)		10.33		0.502¥	8.09		0.440¥
Difference means (c vs. d)		-0.03		1.000¥	0.08		1.000¥
Difference means (c vs. e)		18.04		0.063¥	11.41		0.130¥
Difference means (d vs. e)		18.06		0.071¥	11.33		0.150¥

		Dichotomous score			Polytomous score		
**Group**	**N**	**Mean score (SD)**	**F-value**	**p-value**	**Mean score (SD)**	**F-value**	**p-value**
**Diagnosed with cancer (B4b)**				**0.037** [ab]			0.355
a. Yes	551	29.91 (21.31)			46.42 (14.47)		
b. No	125	33.44 (15.82)			47.51 (11.13)		
Difference means (a vs. b)		-3.54			-1.09		
**Having a general practitioner/family physician (C3)**				0.186			**0.017**
a. Yes	626	30.76 (19.98)			47.03 (13.23)		
b. No	54	26.02 (25.33)			40.26 (19.86)		
Difference means (a vs. b		4.73			6.77		
**Need assistance to see an HCP (C5)**				**0.000**			0.994
a. Yes	302	33.86 (15.25)			46.52 (11.88)		
b. No	375	27.67 (23.54)			46.53 (15.52)		
Difference means (a vs. b		6.19			-0.09		

[¥] post-hoc test using Tukey HSD for homogeneous variance with p-value < 0.05 indicates a significant difference
[§] post-hoc test using Games-Howell for heterogenous variance with p-value < 0.05 indicates a significant difference
